# Reentrant Condensation of Polyelectrolytes Induced by Diluted Multivalent Salts: The Role of Electrostatic Gluonic Effects

**DOI:** 10.1021/acs.biomac.4c01037

**Published:** 2024-10-21

**Authors:** Huaisong Yong

**Affiliations:** † Department of Molecules & Materials, MESA+ Institute, University of Twente, AE 7500 Enschede, the Netherlands; ‡ Institute Theory of Polymers, Leibniz-Institut für Polymerforschung Dresden e.V., D-01069 Dresden, Germany; § School of New Energy and Materials, Southwest Petroleum University, Chengdu 610500, China

## Abstract

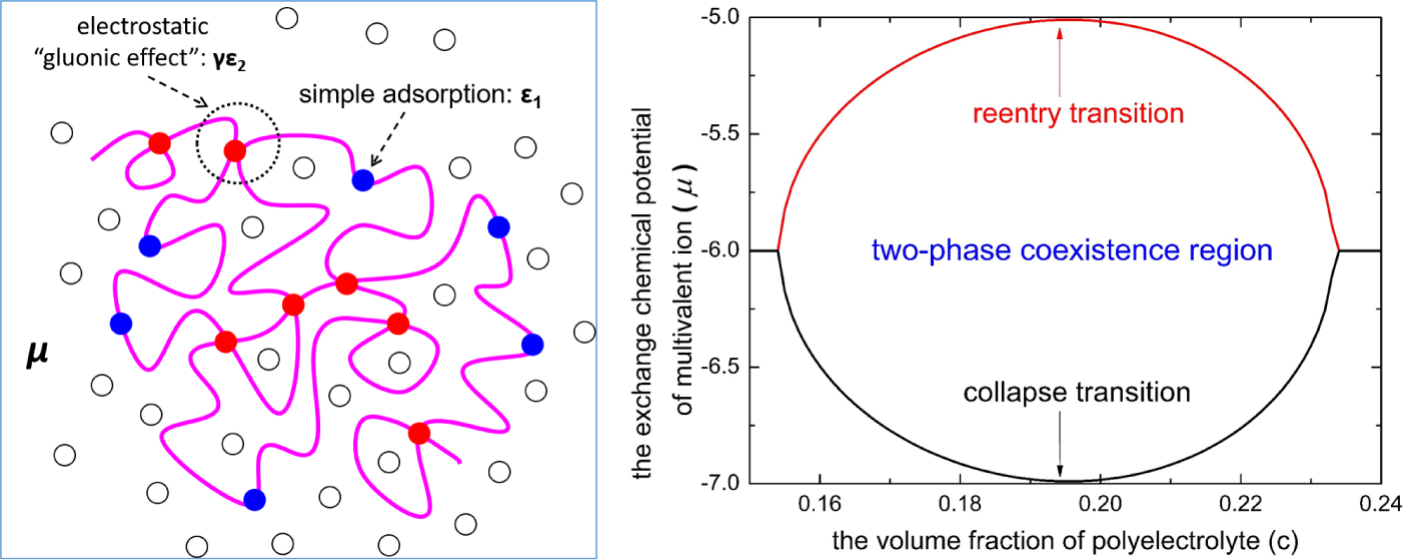

We explore the reentrant condensation of polyelectrolytes triggered by multivalent salts, whose phase-transition mechanism remains under debate. We propose a theory to study the reentrant condensation, which separates the electrostatic effect into two parts: a short-range electrostatic gluonic effect because of sharing of multivalent ions by ionic monomers and a long-range electrostatic correlation effect from all ions. The theory suggests that the electrostatic gluonic effect governs reentrant condensation, requiring a minimum coupling energy to initiate the phase transition. This explains why diluted salts with selective multivalency trigger a polyelectrolyte phase transition. The theory also uncovers that strong adsorption of multivalent ions onto ionic monomers causes low-salt concentrations to induce both collapse and reentry transitions. Additionally, we highlight how the incompatibility of uncharged polyelectrolyte moieties with water affects the polyelectrolyte phase behaviors. The obtained results will contribute to the understanding of biological phase separations if multivalent ions bound to biopolyelectrolytes play an essential role.

## 1 Introduction

**Figure 1:**
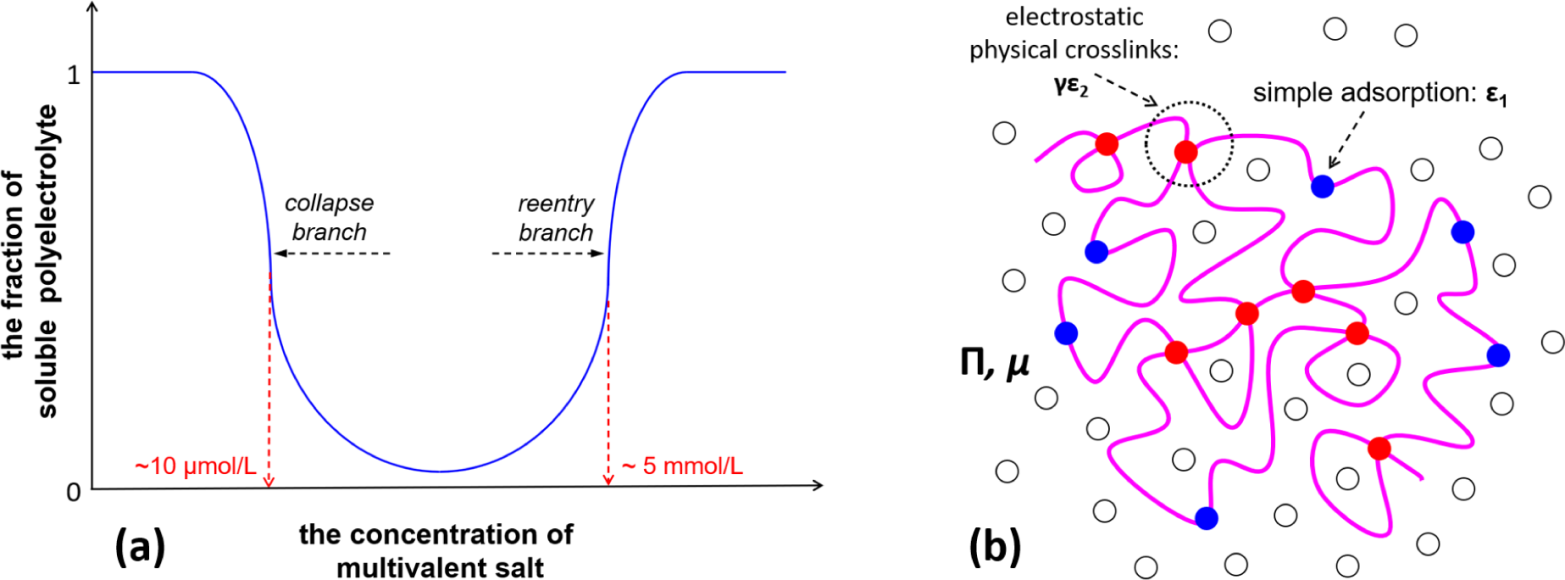
(a) Sketch description of the reentrant condensation of polyelectrolytes induced by diluted multivalent salts. In the figure, the typical values of the multivalent salt concentration for collapse and reentry transitions are quoted from refs.^8,11^ Notice that the reentrant condensation is not necessary to be symmetric with respect to multivalent salt concentrations. (b) A sketch description of preferential adsorption of multivalent ions by ionic monomers (filled blue circles) and forming “physical cross-links” between ionic monomers by sharing multivalent ions (filled red circles). Here, the role of the multivalent ion is just like a glue to bind different ionized monomers. In the figure, the pink lines represent polymer chains and open black circles represent background ions in polyelectrolyte solutions.

When polyelectrolytes mix with multivalent salts, rich phase transitions can occur by variations in salt compositions, temperature, and polymer concentration, which have attracted growing attention in the past decades.^1−7^ A particular scenario is the reentrant condensation of polyelectrolytes such as proteins in the aqueous solution of small multivalent salts, as sketched in Figure 1a, where the collapse transition occurs when a rather small concentration of multivalent counterions is introduced (such as tripolyphosphate, Fe^3+^, and Y^3+^), and the reentry transition of the precipitated proteins occurs when a minor excess of the multivalent counterions is further added.^8−11^ Clarifying the function of diluted multivalent salts in this reentrant condensation is pivotal for a better understanding of liquid–liquid phase separation in biological systems if multivalent ions bound to biopolyelectrolytes (such as RNAs^12^ and proteins^13^) play an essential role.^1,3,6,14−16^

A remarkable aspect of such a reentrant transition is that (as sketched in Figure 1a) the concentration of overall added multivalent salt is quite low (usually on the order of 5 mmol/L) when the reentrant condensation occurs,^8,11^ which implies that the effect of electrostatic screening actually plays a trivial role, since the Debye length of electrostatic screening is comparable with the size of an isolated polyelectrolyte chain or even larger in this case.^17−19^ This peculiar feature indicates that classical theories,^17,20−27^ which are based on the effect of counterion condensation^20,24−27^ and the effect of electric dipole–dipole attraction,^17,21^ as well as the coacervation effect of polyanions with polycations,^22,23^ remain challenging to understand/analyze the reentry branch of polyelectrolyte condensation in diluted multivalent salt solutions^8,11^ as illustrated in Figure 1a. In general, these classical theories^17,20−27^ rely heavily on the effect of electrostatic screening to explain the reentry branch of polyelectrolyte condensation where a necessary condition is the massive addition of small salts.

Another remarkable aspect of such reentrant transition (as sketched in Figure 1a) is that the *ad hoc* pH effect merely caused by hydrolysis of some multivalent metal salts is not a primary factor in regulating phase behaviors of certain biopolyelectrolytes (such as proteins^11,28,29^). This feature implies that it is possible to exclude *ad hoc* effects and consistently understand both the collapse and reentry branches of polyelectrolyte condensation in dilute solutions of multivalent salts. Compared with classical theories,^17,20−27^ a consistent and intuitional way to explain the reentrant transition (as illustrated by Figure 1a) can be as follows: We consider an adsorption process, where ionic monomers are the substrate for ions. When there is a higher affinity of ionic monomer with multivalent ions compared to monovalent ions, for example, in the system of poly(acrylic acid) /sodium ion/rare-earth metal ions,^30^ multivalent ions prefer to adsorb on ionic monomers and form coordination complexes as sketched in Figure 1b. Notwithstanding, if we solely take into account pairwise-like interactions for preferential adsorption between multivalent ions and ionic monomers, linear polymers are 1D substrates for ions. From a theoretical consideration, this situation can be taken into account in formal analogy with the 1D-Ising model.^31,32^ Therefore, one should not expect a phase transition merely according to the simple exchange effect of ion preferential adsorption.

Thus, it is necessary to further account for an additional attraction between multivalent ions and ionic monomers. An intuitive but quite natural way to realize the attractive interaction is the sharing of a multivalent ion by several ionic monomers, i.e., forming electrostatic physical cross-links between ionic monomers by sharing multivalent ions as sketched in Figure 1b. Such electrostatic “cross-linking attraction” also enhances the adsorption of multivalent ions by increasing the adsorption energy, which was corroborated in detail by recent all-atom simulation studies of Wang and coworkers^33,34^ on the phase behaviors of poly(acrylic acid) and sodium polyacrylate in aqueous solutions of calcium salts. To reach more binding with multivalent ions, a collapsed conformation can be preferred when the attraction strength is strong enough, even if the entropy of the chain conformations is reduced. This introduces a coupling between adsorption and attraction and in turn leads to an effective nonlinear attractive interaction between ionic monomers beyond the pairwise-like interaction. The physics picture behind this approach implies that the electrostatic “cross-linking attraction” is a kind of short-range interaction, which cannot be addressed properly by classical theories,^20,24−27^ such as the “double screening theory”,^25,35^ that mainly consider the pairwise-like electrostatic interaction due to the long-range correlation of all ions.

In the past 30 years, theoretical formalisms^36−49^ in the spirit of the effect of electrostatic “cross-linking attraction” between ionic monomers, such as concepts of “ion bridges”^42,43^ and “complexations”,^48^ as well as “short-range attraction”,^40^ have significantly promoted the understanding of phase behaviors of polyelectrolytes in the presence of multivalent salts. However, it is necessary to point out that they remain absent from addressing the fact that polyelectrolytes can only show phase transition in the dilute solution of salts with selective multivalency.^50−55^ For example, bovine serum albumin proteins show phase transition in aqueous solutions of trivalent and tetravalent salts but not in aqueous solutions of divalent salts.^52^ In addition, most of previous theoretical formalisms^36,49^ did not pay enough attention to the influence of nonelectrostatic interactions such as hydrophobic interaction, which indeed plays an important role in regulating phase transition of polyelectrolyte as indicated by recent experimental investigations on solution properties of proteins^56−60^

In parallel, a quite similar situation exists in the research of the cononsolvency effect of polymers (a typical reentrant condensation of polymers), where the binary mixtures of two good solvents can be poor solvents of the same polymer.^61^ With analogy, we note that the concept of cosolvent-assisted “physical cross-linking” effect was proposed by Sommer^62,63^ to explain cononsolvency effect, which can be extended to consistently rationalize the current level of research^1−7^ on the collapse and reentry transitions of polyelectrolytes in dilute solutions of small multivalent salts.^a^

Bearing these points in mind, in this work, by viewing the multivalent ion as a kind of charged “gluonic cosolute”,^62,63^ we constructed a simple but effective mean-field model which can consistently rationalize the essential features of the reentrant condensation including the phase diagram of polyelectrolyte. We separate the electrostatic effect into the short-range electrostatic gluonic effect due to sharing multivalent salt ions between ionic monomers and the nonassociative pairwise-like electrostatic effect arising from the long-range correlation of all ions. This approach allows us to uncover that the electrostatic gluonic effect dominates the reentrant transition of the polyelectrolyte induced by diluted multivalent salts. The analytical solution of the model indicates that a minimum coupling energy for the electrostatic gluonic effect is essential for phase transition to occur, which, for the first time, explains the enigmatic observation that polyelectrolytes can only show phase transition in a dilute solution of salts with selective multivalency. The model also unveils that strong adsorption between the ionic monomers and multivalent ions can be at the origin of the peculiar phenomenon in which rather low concentrations of multivalent salts trigger both collapse and reentry transitions.

In the remainder of this article, the physical model for the polyelectrolyte solution in terms of the free energy is constructed in Section 2. Its analytical solution will be considered in detail in Section 3, where we will outline some general consequences of the model, and a simplified phase diagram of the polyelectrolyte solution will also be discussed in this section. Finally, the applicability of the model is discussed in Section 4 with concluding remarks.

## 2 Methods and Model

In this section, we construct the physical model for the polyelectrolyte solution in terms of the Gibbs free energy and clarify its physics foundation.

For simplicity but without losing generality, as sketched in Figure 1b, we consider a flexible polyelectrolyte with monovalent ionic monomers and monovalent counterions when no salt is introduced. We denote *N* as the number of monomers in a polyelectrolyte chain and *a* as the size of each monomer along the direction of the polymer backbone. The charged monomers are distributed randomly on the polyelectrolyte chain, and their fraction is denoted by *p*. Then, the average distance between neighboring charged monomers is *a*/*p*. There is an effective saturated charge density for polyelectrolyte chains due to the repulsive correlation effect in the dissociation of neighboring ionic monomers.^18^ The effective saturated charge density is determined by the condition that polyelectrolyte does not undergo counterion condensation:^20,24−27^
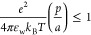
1Here, *e* is the electron charge, ε_w_ is the absolute dielectric permittivity of water, *k*_B_ is the Boltzmann constant, and *T* is the thermodynamic temperature. Because the Bjerrum length *l*_B_ = *e*^2^/(4πε_w_*k*_B_*T*) is about 0.74 nm for water and *a* is about 0.25 nm for vinyl polymers (the length of two carbon–carbon single bonds), we see that *p* ≤ *a*/*l*_B_ ≈ 1/3, which is usually satisfied by amphiphilic polyelectrolyte such as proteins.^64,65^ On account of this consideration and to simplify our following discussions, in this study, we focus on polyelectrolyte whose charge density is under the effective saturated charge density, i.e., the case of *p* ≤ *a*/*l*_B_ ≈ 1/3.

Here and in the following, let us consider the free energy per unit of volume for an incompressible system if not otherwise noted specifically. The volume unit is given by the size of the solvent molecules in the spirit of the classical Flory–Huggins lattice model.^66,67^ For simplicity but without losing generality, we assume that the sizes of the ionized monomers, counterions, salt ions, and solvent molecules are the same. The added multivalent salt has the chemical formula XZ_*x*_, where X is the multivalent cation with the valence +*x* and Z is the monovalent anion. The overall volume fraction of monovalent ionic monomers and neutral monomers is denoted by *c*, and then, the volume fraction of monovalent counterions is *pc*. We denote the volume fraction of multivalent cations X as *c*_X_, and then, the volume fraction of monovalent anion Z is *xc*_X_.

The Gibbs free energy, in general, consists of many terms. The most common term is the contribution from the mixing of polymer with solvent and added multivalent salt, *G*_sol_. Within the mean-field approximation, the free energy of *G*_sol_ is given by
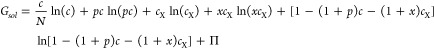
2

As sketched in Figure 1b, here we separate the system into two parts: the polymer chains with their enclosed solvents and small ions and the bulk without polymer chains. The bulk is just described by its osmotic pressure (Π) acting on the first part. If the volume of the polymer coils changes, then there is the mechanical work against the external pressures involved in the free energy change. This will become important when we minimize the free energy with respect to the monomer concentration (*c*). Here and in the following, we consider energies in units of *k*_B_*T* if not otherwise noted specifically.

Since the exchange of counterions only occurs on the ionized monomers, the adsorption free energy per unit of volume owing to the mixing of multivalent and monovalent cations on the polymer chains is therefore given by *G*_ads_:
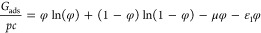
3Here, φ is the fraction of ionic monomers occupied preferentially by multivalent cations. ε_1_ denotes the preferential-adsorption strength of one multivalent cation with respect to the polyelectrolyte, which stems primarily from ionic-bond interactions or electric dipole interactions and is often not small (on the order of about 5*k*_B_*T* for the strength of an ionic bond in water at low-salt concentration^68^ and on the order of about 10*k*_B_*T* for the strength of electric dipole interaction^69^). μ represents the chemical potential of exchanging a monovalent cation by a multivalent cation on the polymer chains, which scales as μ ∼ ln(*c*_X_) if *c*_X_ is very small.

We consider the associative electrostatic attraction *G*_attr_ between ionized monomers caused by forming a bridge due to multivalent cations, where the bridge is a kind of short-range attractive interaction as indicated by previous experiments^60^ and proven numerically by the Gaussian renormalized fluctuation theory recently.^40^ As sketched in Figure 1b, here the role of the multivalent cation is just like a glue to bind different ionized monomers, which is in analogy to the concept of “gluonic cosolvent” proposed by Sommer^62,63^ to explain cononsolcency effects.^61^ Statistically, the term of *G*_attr_ is proportional to the average probability that a given ionized monomer (φ or 1 – φ states) is in contact with an ionized monomer of the other state (1 – φ or φ). Here, the state of the ionized monomer is defined by either having adsorbed a multivalent cation (with probability φ) or being empty (only surrounded by solvent and monovalent cation, with probability 1 – φ). The strength of this additional short-range attraction (electrostatic gluonic effect) is given by γε_2_, which takes into account the bridging efficiency of the molecular matching^50,51^ between the ionized monomer and the multivalent ion by the numerical coefficient γ *≠* 0. Since the electrostatic gluonic effect only occurs among ionized monomers, the attraction energy owing to the electrostatic gluonic effect is therefore given by the mean-field approximation:
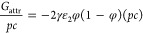
4

The ionic-bond features of adsorption and attraction imply that ε_1_ ≈ ε_2_ are due to the chelation of multivalent ions by ionic monomers. A physical boundary condition is embedded in *G*_attr_ that is both φ → 0 and φ → 1, leading to the vanishing of *G*_attr_ → 0 since this condition corresponds to the fact of reentrant transition that polyelectrolyte must be miscible within aqueous solutions with low and high concentrations of multivalent salts, respectively. Notice that another physical boundary condition can be embedded in *G*_attr_ by γ = (*x* – 1)γ_0_ with γ_0_*≠* 0, which is monovalent + *x* = 1, leading to the vanishing of *G*_attr_ → 0 since this condition corresponds to the fact that the dilute solution of monovalent salts cannot lead to the phase transition of polyelectrolytes.

A basic characteristic of the statistical formulation of eq 4 is that it shows the number of bridges per ionic monomer first increases and then declines with the amount of adsorbed multivalent ions on polyelectrolyte chains (φ), i.e., ∝2φ(1– φ)(*pc*), which was confirmed qualitatively by recent all-atom simulation results reported by Wang and coworkers.^33,34^ Another characteristic of eq 4 is that it implies the strength of the electrostatic gluonic effect (γε_2_) declines with increasing temperature and in turn induces a weaker phase transition, which aligns with recent fluorescence experiments on phase behaviors of protein solutions in the presence of multivalent salts.^70,71^ The preferential adsorption of multivalent ions on polyelectrolyte chains with the physical cross-linking of ionic monomers inevitably leads to overcharging and charge inversion^72,73^ of polyelectrolyte chains at certain concentrations of multivalent salts, which implies that the free energy of polyelectrolyte solution must have minimum(s) or symmetry breaking(s) around these salt concentrations.^33,34,40,74,75^ We note that this fact is reflected by the statistical construction of eq 4 with a minimum and a symmetry breaking at φ = 1/2, which corresponds to the phase transition from soluble to collapsed states (including of both collapse and reentry transitions), and this viewpoint will become clear in detail by the analytical analyses from Sections 3.1 and 3.2. Because of this feature, the fraction of polyelectrolyte chains adsorbed preferentially by multivalent ions (φ) is also the order parameter for the phase transition in our model.

Besides the short-range electrostatic gluonic energy considered by eq 4, there also exists the nonassociative pairwise-like electrostatic interaction due to the long-range correlation of all ions. This long-range correlation has been well-understood by various classical polyelectrolyte theories.^20,24−27^ In our study, for mathematical simplification but without compromising on the general physical conclusions, we take care of the nonassociative pairwise-like electrostatic interaction in the low-salt limit by the classical “double screening theory”^25,35^ as *G*_DS_:
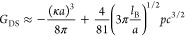
5where the inverse Debye screening length *κ* is given by (*κa*)^2^ = 4π(*l*_B_/*a*)(*pc* + (*x*^2^ + *x*)*c*_X_). The first term of eq 5 stems from the nonassociative attraction correlation of all small ions, which is approximated by the Debye–Hückel theory for the isothermal–isobaric ensemble (NPT ensemble).^76^ The second term is from the fluctuation contribution of ionized polymer chains, which is always positive in virtue of the intrinsically electrostatic repulsion between ionized monomers.^77^

The free energy contribution by the formalism of eq 5 is negative for experimental values of *l*_B_/*a*, which aligns with classical polyelectrolyte theories.^20,24−27^eq 5 is valid when the ionic strength of the added small salt is low, strictly valid if on the order of about 5 mmol/L and qualitatively valid if in a few orders of 200 mmol/L,^76,78^ which usually is also the regime for the occurrence of the reentrant condensation of polyelectrolyte.^8,11^ In salt solutions, salt ions usually coordinate with polymer backbones with their hydration shells.^79,80^ It is thus expected that both the electrostatic adsorption and attraction are not permanent and have a dynamic-bond feature, which was confirmed by recent all-atom simulation studies of Wang and coworkers^33,34^ on phase behaviors of poly(acrylic acid) and sodium polyacrylate in aqueous solutions of calcium salts. To simplify analytical calculations but without losing generality to get key physical conclusions for the current research, the effect of charge neutralization has been accordingly ignored in the formulation of the free energy of electrostatic interaction.

The monomers of polyelectrolyte chains are composed of both charged and uncharged moieties. This is also true for charged monomers. The energy of nonelectrostatic excluded-volume interactions between solvent (water) and the charge neutral part of monomers is given by the classical Flory–Huggins^66,67^ formalism *G*_FH_:
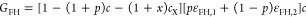
6where ε_FH,1_ is the Flory–Huggins parameter between solvent and charged monomers and ε_FH,2_ is the Flory–Huggins parameter between solvent and uncharged monomers. We point out that the hierarchy of non-Coulomb interaction (ε_FH,1_ ≤ ε_FH,2_) usually exists between charged and uncharged monomers.

The total free energy per volume unit is simply considered as
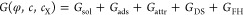
7

## 3 Results and Discussion

### 3.1 The Minimum Coupling Energy for the Short-Range Electrostatic Gluonic Effect in Phase Transition

The polymer behavior of the maximum collapsed state can be understood analytically. Based on eq 4, the maximum coupling is achieved at the point φ = 1/2. Thus, close to the half-occupied regime at φ = 1/2, a maximum collapsed state of the polymer chains can be expected. Because we are interested in the case of a very diluted solution of multivalent salt (*c*_X_ → 0), here we ignore the influence of multivalent salt in eqs 2, 5, and 6 by setting *c*_X_ = 0. This assumption allows us to highlight the gluonic effect between polymer chains in the same spirit as the treatment in refs.^62,63^ For the case of maximum coupling (φ = 1/2) with the volume fraction of multivalent ions at *c*_X_ → 0, which results in both the chemical potential μ and the osmotic pressure Π approaching their limiting values μ_0_ and Π_0_, respectively. This in turn mathematically casts our model to a canonical ensemble-like model for the polymer solution:
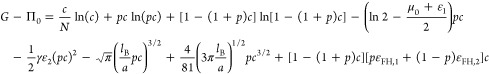
8Here, the canonical ensemble-like free energy is given by *G* – Π_0_.

By eq 8, we can roughly estimate a minimum coupling energy (γε_2_) for the electrostatic gluonic effect that is necessary for a phase transition. This can be realized by determining the boundary condition for the spinodal decomposition of polyelectrolyte solution, which is given by d^2^(*G* – Π_0_)/d*c*^2^ = 0 in analogy to the framework of the canonical ensemble and follows closely with ref.:^62^
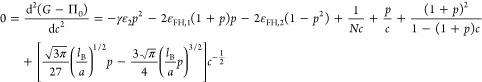
9The computing methodology behind this approach can be found in Appendix A (Supporting Information). Here, we define the overall effective interaction parameter 2χ_0_ ≡ γε_2_*p*^2^ + 2ε_FH,1_(1 + *p*)*p* + 2ε_FH,2_(1 – *p*^2^). The critical or minimum value of it to allow phase transition is given by d(*2*χ_0_)/d*c* = 0:
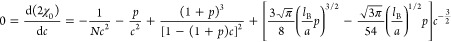
10There exists no exact explicit analytical solution for *c* in eq 10, but a good approximation solution can be obtained by ignoring the fourth term since it is much smaller than the sum of other terms within experimental values of the parameter *l*_B_/*a*. For the critical point of the polyelectrolyte collapse, we obtain an approximation for the critical volume fraction of monomers *c* at
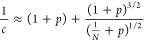
11The exact solution of eq 10 is recovered by eq 11 when the parameter *p* approaches zero. One can improve the analytical solution of eq 10 by using the method of fixed-point iteration^81,82^ with the initial value defined by eq 11. However, the approximate solution of eq 11 is sufficient to deduce the key features of the overall effective interaction parameter χ_0_ without compromising the physical conclusions.

By insertion of eq 11 into eq 9, we get an estimation for the critical or minimum value of χ_0_:
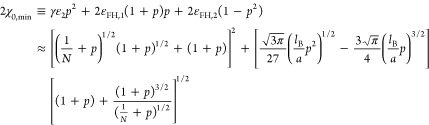
12By setting *p* = 0 in eq 12, we recover the boundary condition for the spinodal decomposition of an uncharged polymer solution, i.e., . Then, we get the minimum of γε_2_ for the case of very long polyelectrolyte chain (*N* → ∞) by eq 12:
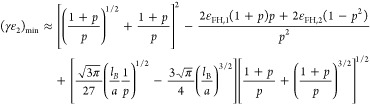
13Notice that the minimum coupling energy for the electrostatic gluonic effect (γε_2_) estimated by eqs 12 and 13 is unrelated to model parameter ε_1_. This is an expected result, as we have pointed out that there is no phase transition merely according to the simple exchange effect of ion preferential adsorption (ε_1_) in formal analogy with the 1D-Ising model (see the third paragraph of Introduction).

**Figure 2:**
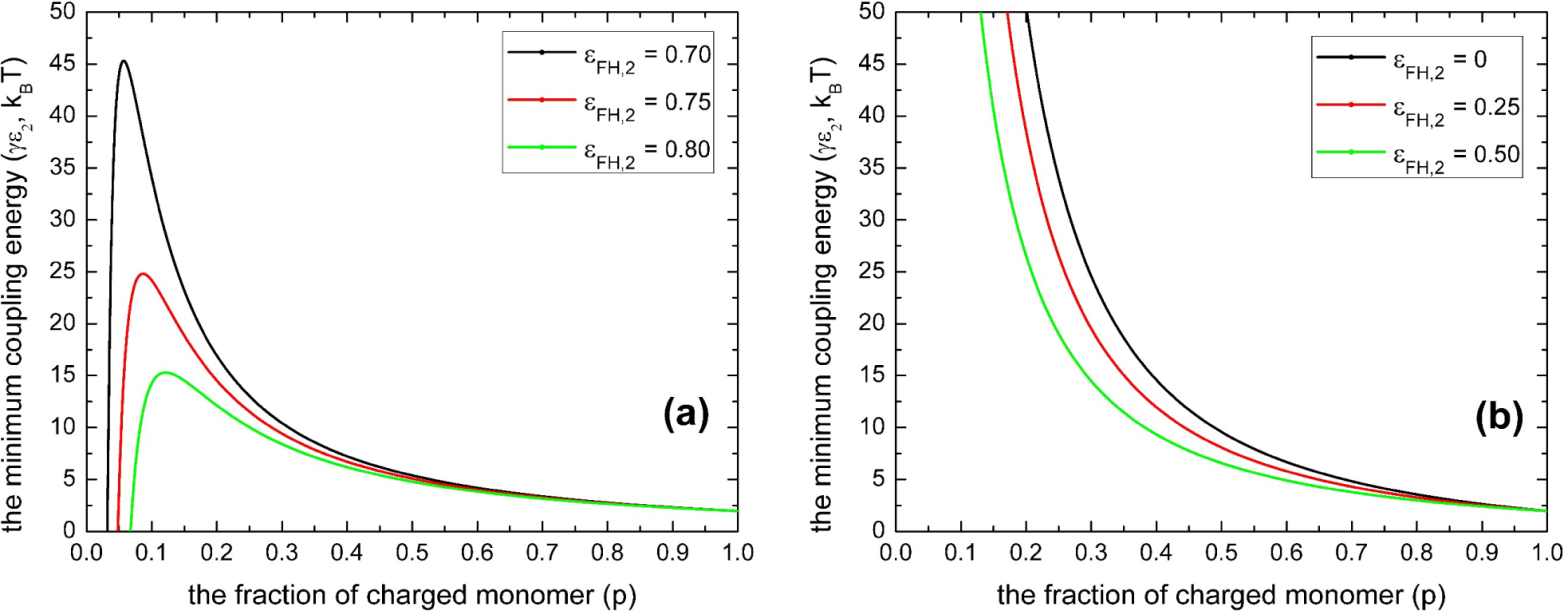
Minimum coupling energy (γε_2_) with respect to the fraction of charged monomer (*p*) according to eq 13 for typical values of the parameter ε_FH,2_ with *l*_B_/*a* = 2, ε_FH,1_ = 0.45, and *N* → ∞ for (a) ε_FH,2_ > 1/2 and (b) 0 ≤ ε_FH,2_ ≤ 1/2.

**Figure 3:**
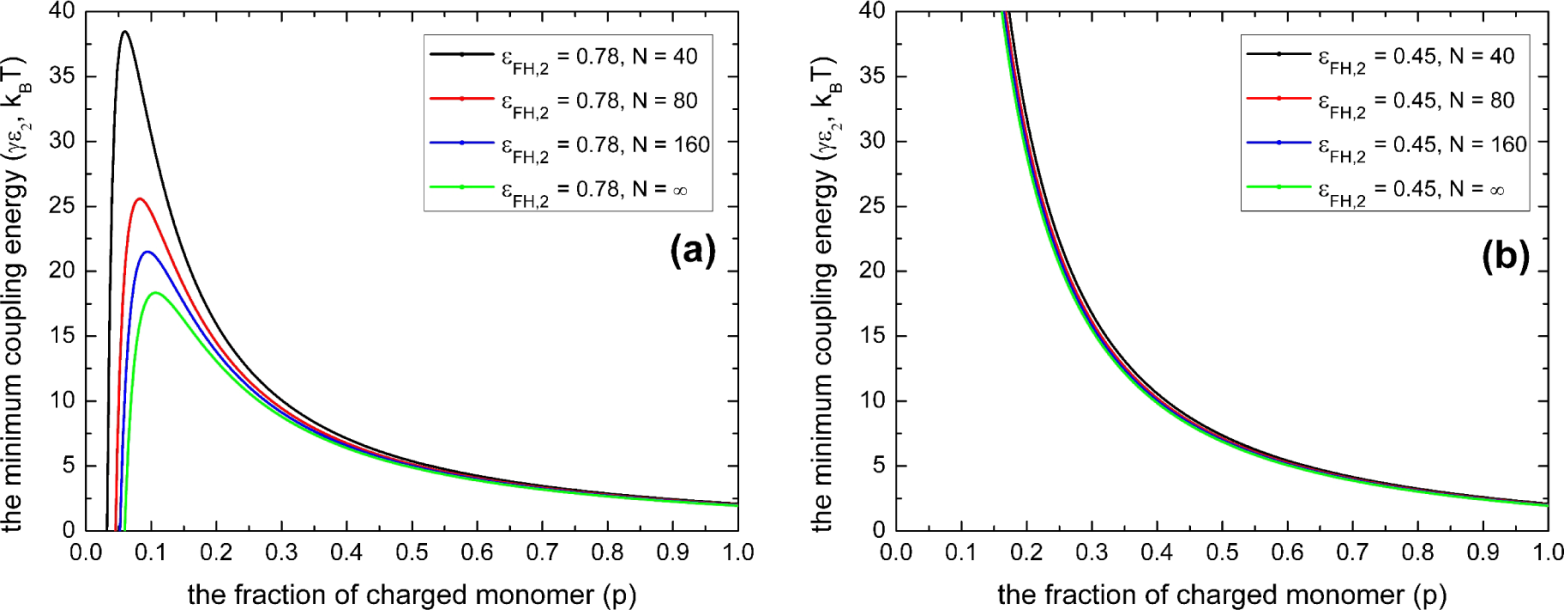
Minimum coupling energy (γε_2_) with respect to the fraction of charged monomers (*p*) according to eq 12 for various polyelectrolyte chain lengths (*N*) with *l*_B_/*a* = 2 and ε_FH,1_ = 0.45 for (a) ε_FH,2_ = 0.78 and ε_FH,2_ > ; panel (b) for the case of and ε_FH,2_ = 0.45 and 0 ≤ ε_FH,2_ ≤ .

A remarkable feature of the constructions of eqs 12 and 13 is that no matter the values of ε_FH,1_ ≥ 0 and *l*_B_/*a* ≥ 0, there is an unique local maximum of (γε_2_)_min_ when ε_FH,2_ is larger than , but no local maximum of (γε_2_)_min_ exists when 0 ≤ ε_FH,2_ ≤ . This feature is coincidental with the well-known Θ-condition for uncharged polymers. Thus, we can already see that the minimum coupling energy (γε_2_)_min_ for the electrostatic gluonic effect to lead to a phase transition is largely regulated by the solvent quality (parameter ε_FH,2_) for the uncharged part of the polyelectrolyte. For most amphiphilic polymers in aqueous solutions, the value of *l*_B_/*a* is on the order of unity and *l*_B_/*a* ≲ 3 for vinyl polymers. As exemplars of *l*_B_/*a* = 2 and ε_FH,1_ = 0.45 are shown in Figures 2 and 3, the unique local maximum of (γε_2_)_min_ is regulated by the well-known poor-solvent condition for the uncharged monomers, i.e., .

As indicated in Figure 3a, given the condition of ε_FH,2_ > , the necessary value of coupling energy (γε_2_)_min_ for phase transition to occur can be influenced drastically by the chain length (*N*) of polyelectrolytes. In contrast, it is not sensitive to the polyelectrolyte chain length provided that 0 ≤ ε_FH,2_ ≤ , as shown in Figure 3b. This feature may be critical for understanding the reentrant condensation of some amphiphilic proteins since the molecular weight of proteins usually is not very large. By a comparison of Figures 2a,b, as well as by a comparison of Figures 3a,b, we can see that it is possible to have phase transition for polyelectrolytes when the solvent (water) quality is good for the uncharged building blocks. However, the necessary value of coupling energy (γε_2_)_min_ for phase transition to occur is much higher for this situation. This is the case^5,33,34^ such as reported recently by Wang and coworkers^33,34^ for poly(acrylic acid) and sodium polyacrylate in aqueous solutions of calcium salts, where one calcium cation can coordinate as many as four or more ionic acrylate monomers even though the valence of calcium cation is two. These predictions of our model are in line with the fact that nonelectrostatic interactions, such as hydrophobic interaction, play an important role in regulating the phase transition of polyelectrolytes, as indicated by recent experimental research on solution properties of proteins.^56−60^

### 3.2 The Concentration-Dependent Effective Flory–Huggins χ Parameter

In order to find the equilibrium state of the polyelectrolyte phase with respect to the bulk solvent phase, first minimize the free energy *G*(φ, *c*, *c*_X_) with respect to the adsorption fraction of multivalent cation φ. Then, the replacement of the solution φ(μ, *c*, *c*_X_) into the free energy leads to an effective free energy for the polyelectrolyte, where the effect of multivalent ions is mapped onto an effective monomer–monomer interaction, which will depend on the concentration of multivalent ions. Finally, the minimization of the free energy per monomer, i.e., *G*(φ, *c*, *c*_X_)/*c* instead of *G*(φ, *c*, *c*_X_), with respect to the monomer concentration (*c*), leads to the equilibrium solution of the model. The computing methodology behind this minimization approach can be found in Appendix B (Supporting Information) and follows closely with refs (62), (63), (83), and (84).

Because we are interested in the case of very negative values of μ, which corresponds to the very diluted solution of multivalent salt (*c*_*X*_ → 0), this means that in the calculation, we can approximate φ(μ, *c*, *c*_X_ → 0) by φ(μ, *c*) and approximate *G*(φ, *c*, *c*_X_ → 0)/*c* by *G*(φ, *c*)/*c*. Physically speaking, this approximation can also be elucidated in that we highlight the adsorption and attraction effects of added small ions near/on polyelectrolyte chains and ignore their own nonessential mixing effects if these added small ions are far away from polyelectrolyte chains. This approach will avoid heavy calculations without losing generality to obtain key physical conclusions.

Equation 4 implies that the maximum contraction of polymer chains is reached at the symmetry breaking point φ = 1/2, where the electrostatic gluonic effect reaches its maximum, which corresponds to the phase transition from soluble to collapsed states (including both collapse and reentry transitions). This peculiar feature of the model will significantly simplify our analytical calculations. We introduce a perturbation (δ) from the half occupation of the chain by the multivalent ion according to
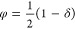
14This perturbation approach follows similarly with refs (62), (63), and (83). With the expansion of δ-containing terms in the logarithm function up to the accuracy of square terms (δ^2^ under the constraint of |δ^3^| ≪ 1 and ignoring constant terms, we obtain
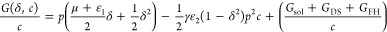
15with
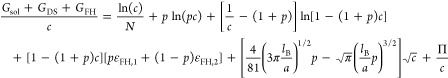
16

Minimizing the free energy in eq 15 with respect to δ yields
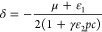
17Resubstituting eq 17 into eq 15, we obtain
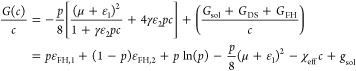
18with the effective Flory–Huggins parameter χ_eff_:
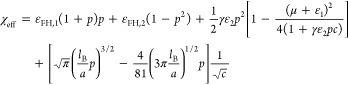
19and the entropic term of free energy per monomer *g*_sol_:
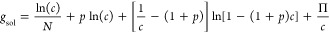
20In eq 19, we separated the effective Flory–Huggins parameter χ_eff_ into three components. The first and second terms of the right hand of eq 19 are the contribution of nonelectrostatic excluded-volume interaction, the third term is the contribution of electrostatic gluonic effects between ionic monomers due to sharing of multivalent salt ions, and the fourth term is the contribution of the nonassociative electrostatic pairwise-like interaction arising from the correlation of all ions. The effective Flory–Huggins parameter χ_eff_ reaches its maximum when the value of (μ + ε_1_)^2^ is close to zero, which corresponds to the optimally loaded state of polyelectrolytes with multivalent ions where the concentration of polyelectrolytes in the condensed polymer phase reaches its maximum. This characteristic can be further observed by the features of the spinodal phase diagrams discussed in the following subsection.

The above-constructed χ-function is a function of the square of the chemical potential μ, which indicates that χ corresponds to two values of μ and thus essentially captures the reentrant signature of polyelectrolyte condensation at lower and higher salt concentrations. In contrast, the reentrant signature of polyelectrolyte condensation cannot be well-understood by the classical polyelectrolyte theories,^17,20−27^ which predict that the constructed effective Flory–Huggins χ parameter becomes monotonically larger as the concentration of added salt is increased, or does not change when the concentration of added salt is beyond a certain threshold value. From eq 19, we point out that the reentrant signature of polyelectrolyte condensation is controlled by the electrostatic gluonic effect, since only this effect is nonmonotonic with respect to the salt concentration.

### 3.3 The Spinodal Phase Diagrams

In order to discuss the phase transition, the pressure isotherm Π(*c*, μ; *N*, *l*_B_/*a*, *p*, ε_1_, γε_2_, ε_FH,1_, ε_FH,2_) can be calculated from eq 18 by ∂(*G/c*)/*∂c* = 0, which leads to
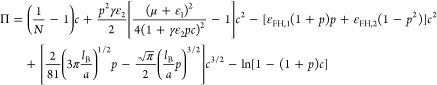
21By setting *p* = 0 in eq 21 for uncharged polymer solution, with ∂Π/∂c = 0 and the constraint of 0 < *c* < 1, as it should be, we recover the boundary condition for the spinodal decomposition of uncharged polymer solution, i.e., ε_FH,2_ > .

**Figure 4:**
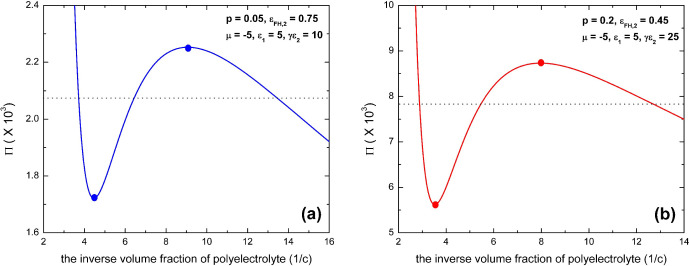
Osmotic pressure of polyelectrolyte solution as a function of the inverse volume fraction of monomers according to eq 21 for the case of *l*_B_/*a* = 2.5, ε_FH,1_ = 0.45, and *N* = 500 for (a) *p* = 0.05, ε_FH,2_ = 0.75, μ = −5, ε_1_ = 5, and γε_2_ = 10 and (b) *p* = 0.2, ε_FH,2_ = 0.45, μ = −5, ε_1_ = 5, and γε_2_ = 25. The coexistence pressures by the Maxwell construction are indicated by the horizontal dotted lines in the figure, and the spinodal points are indicated by filled circles in the figure.

**Figure 5:**
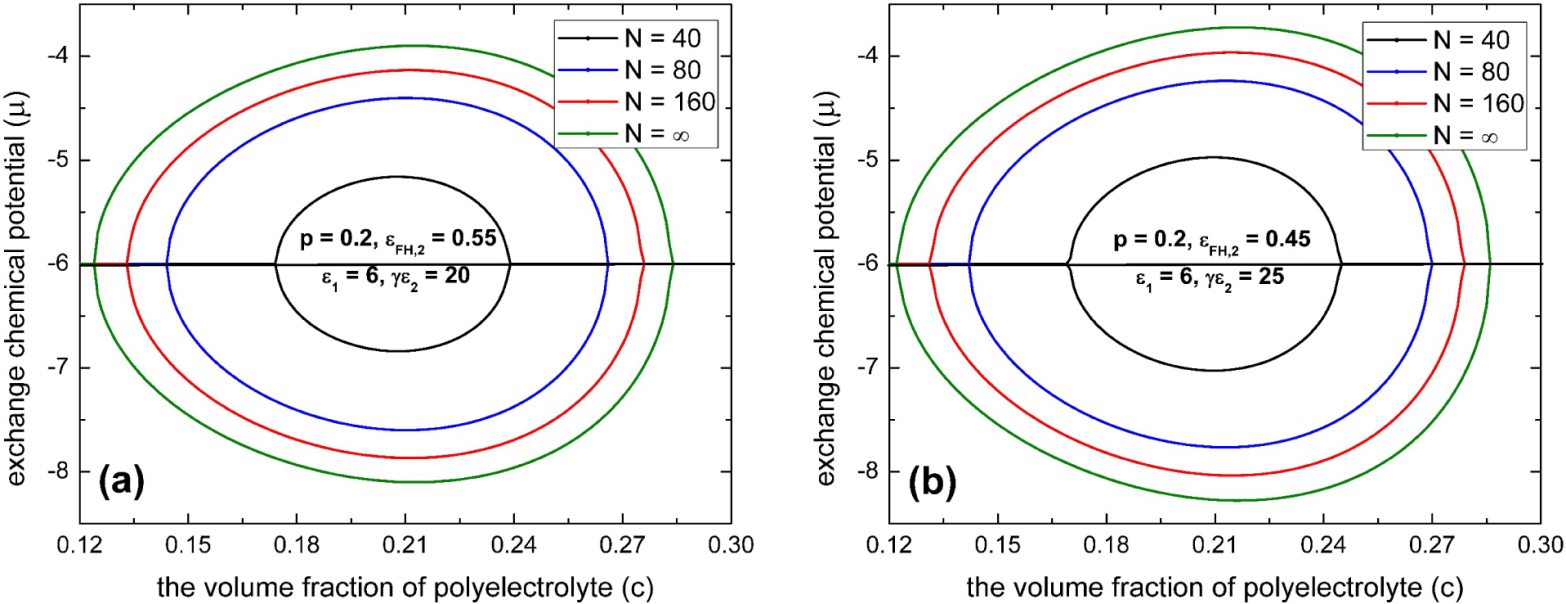
Spinodal phase diagrams of polyelectrolyte in the dilute solution of multivalent salts according to eq 22 for various polyelectrolyte chain lengths (*N*) with *l*_B_/*a* = 2.5 and ε_FH,1_ = 0.45 for (a) *p* = 0.2, ε_FH,2_ = 0.55, ε_1_ = 6, and γε_2_ = 20 and (b) *p* = 0.2, ε_FH,2_ = 0.45, ε_1_ = 6, and γε_2_ = 25.

For the general case of a discontinuous phase transition, the osmotic pressure must display an unstable region of negative compressibility given by ∂Π/∂*c* < 0. In Figure 4, we display two typical examples which show the pressure isotherms for two discontinuous condensation transitions given by eq 21. The coexistence region is defined by the Maxwell construction, as indicated by the horizontal isobaric lines in the figure. This cannot be obtained analytically in an exact way and will not be considered in detail in the following discussions if not otherwise noted specifically. However, we are able to calculate the spinodal analytically at which the solution starts to become unstable, the existence of which is the necessary condition for a discontinuous transition scenario. The spinodal of polyelectrolyte solution is given by ∂Π/∂c = 0 and can be written in the following form:
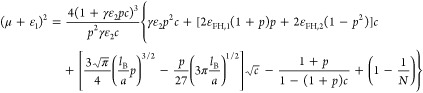
22This defines the spinodal phase diagram in the “μ–*c*” space with the seven parameters *p*, *N*, *l*_B_/*a*, ε_1_, γε_2_, ε_FH,1_, and ε_FH,2_. A basic characteristic of eq 22 is that it predicts that the reentrant condensation of the polyelectrolyte can only occur in a certain range of polymer concentration: There is no phase transition when the concentration of polyelectrolyte is too high or too low, which can be easily read from a spinodal phase diagram, as shown in Figure 5. It is remarkable that this prediction is in line with experimental observations on the phase behavior of proteins in aqueous solutions of multivalent salts.^85,86^

In Figure 5a, we display the spinodal phase diagram in the “μ–*c*” space by eq 22 for a typical case of polyelectrolytes with parameters *p* = 0.2, ε_FH,1_ = 0.45, ε_FH,2_ = 0.55, ε_1_ = 6, γε_2_ = 20, and *l*_B_/*a* = 2.5. In Figure 5b, we display the spinodal phase diagram in the “μ–*c*” space according to eq 22 for a typical case of polyelectrolytes with parameters *p* = 0.2, ε_FH,1_ = 0.45, ε_FH,2_ = 0.45, ε_1_ = 6, γε_2_ = 25, and *l*_B_/*a* = 2.5. The two solutions at the same value of μ correspond to the two extrema of the pressure isotherm, which correspond to a coexistence of condensed and dissolved phases. The region of demixing is closed topologically. For our model, we obtain a symmetric collapse and reentry transition. The lower part of μ defines the collapse transition as indicated by the lower half of the “egg-shape” curve, while the higher part of μ defines the reentry transition. Noticeable is the strong dependence of the chain length on the phase transition. A state that is condensed at a given concentration of multivalent salts can be dissolved if the chain length is reduced. However, in contrast to uncharged linear polymers,^17,19,62,87,88^ our model shows that it is hard to realize a real dilute phase for the reentrant condensation of polyelectrolyte when the charge fraction is not sufficiently low. As indicated in Figure 5, this is particularly noticeable in the limiting case of infinite chain length; there remains no small monomer concentration (*c*) in the dilute phase for small values of (μ + ε_1_)^2^ when phase separation occurs, i.e., close to the optimally loaded state of the polyelectrolyte with multivalent ions where the effective Flory–Huggins parameter χ_eff_ reaches its maximum (see eq 19).

Because of a significant shift of ε_1_ on the left-hand side of eq 22, which is often not small for polyelectrolytes (on the order of about 5*k*_B_*T* for the strength of an ionic bond in water at low-salt concentration^68^ and on the order of about 10*k*_B_*T* for the strength of electric dipole interaction^69^), eq 22 predicts that it melts down when the value of μ is far away from zero, which in particular corresponds to the case of very low concentrations of multivalent salts, such as shown for both the collapsed and reentry branches of the reentrant condensation of polyelectrolytes in Figure 5. Notice that this prediction concurs with existing experimental observations on the reentrant condensation of some proteins in dilute aqueous solutions of multivalent salts^8,11^ where both the collapse and reentry transitions occur at rather small concentrations of multivalent salts.

**Figure 6:**
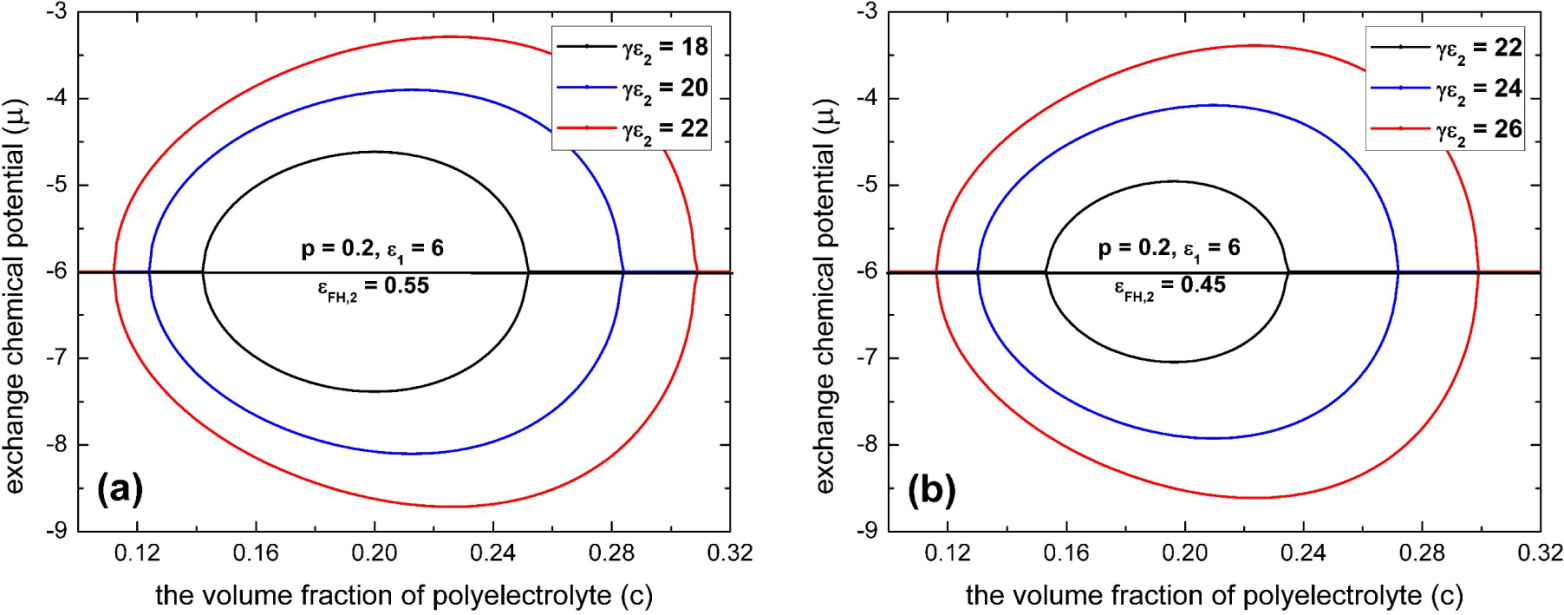
Spinodal phase diagrams of polyelectrolytes in the dilute solution of multivalent salts according to eq 22 for various values of the electrostatic gluonic effect (γε_2_) with infinite chain length (*N* → ∞), *l*_B_/*a* = 2.5, and ε_FH,1_ = 0.45 for (a) *p* = 0.2, ε_FH,2_ = 0.55, and ε_1_ = 6 and (b) *p* = 0.2, ε_FH,2_ = 0.45, and ε_1_ = 6.

In Figure 6, we plot the spinodal phase diagrams in the “μ–*c*” space according to eq 22 for the case of infinite chain length (*N* → ∞) with a moderate fraction of charged monomers. We see that an increase of the electrostatic gluonic effect (γε_*2*_) will shift the coexistence region of collapse transition to lower concentration of multivalent salts and will promote the coexistence region of reentry transition to higher concentrations of multivalent salts, which is in agreement with experimental observations for the phase transitions of both synthetic polyelectrolytes^89−91^ and biopolyelectrolytes^52^ in the presence of multivalent salt. It is worth pointing out that this conclusion is also true when the fraction of charged monomers is low. However, we cannot get this conclusion by the spinodal construction of eq 22. The reason is that the osmotic pressure of spinodal calculated by eq 21 can be negative when the fraction of charged monomers (*p*) is low for the case of ε_FH,2_ > . This scenario is thermodynamically forbidden because a stable osmotic pressure cannot be negative for polymer solutions.^17,92^ Now, the spinodal construction of the phase diagram according to eq 22 breaks down; instead, we can only numerically determine a binodal phase diagram for the case of a small *p*. The details for this situation will be discussed in the next subsection.

### Special Features of the Phase Diagram for the Case of ε_FH,2_*>*

3.4

**Figure 7:**
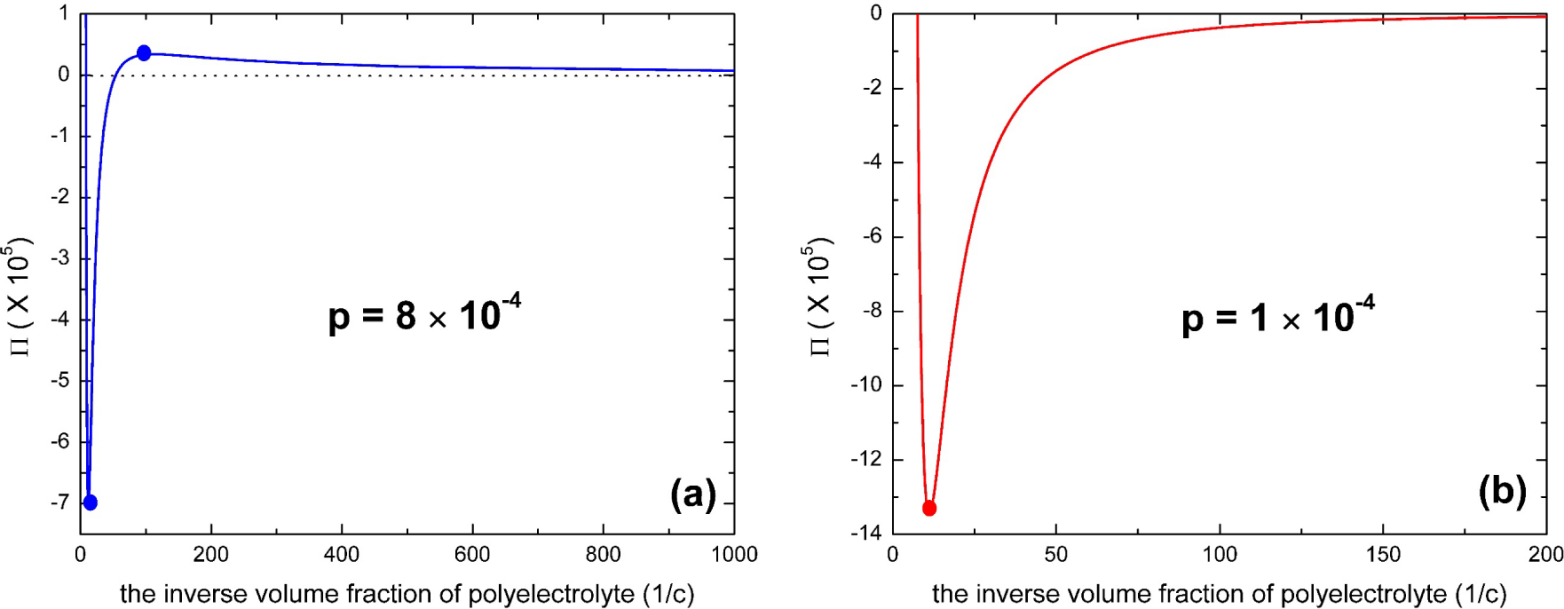
Osmotic pressure of polyelectrolyte solution as a function of the inverse volume fraction of monomers according to eq 21 for the case of infinite chain length (*N* → ∞), *l*_B_/*a* = 2.5, ε_FH,1_ = 0.45, ε_FH,2_ = 0.55, ε_1_ = 5, γε_2_ = 15, and μ = −5 for (a) *p* = 8 × 10^–4^ and (b) *p* = 1 × 10^–4^. The coexistence pressures by the Maxwell construction are indicated by the horizontal dotted line in the figure, and the spinodal points are indicated by filled circles in the figure. Notice that the spinodal with a negative osmotic pressure in the figure is thermodynamically forbidden for polymer solutions.

For the case of ε_FH,2_ > , which particularly corresponds to amphiphilic polyelectrolyte, a spinodal decomposition point of the osmotic pressure may exist (∂Π/∂c = 0) with Π < 0 for some parameter values of *p*, *N*, *l*_B_/*a*, ε_1_, γε_2_, ε_FH,1_, and ε_FH,2_, which is particularly clear in the limiting of large ε_FH,2_ with small *p*. An example of this is shown in Figure 7a. It indicates that by fixing other parameter values, if *p* reaches a critical value at *p* = *p*_0_, the corresponding Maxwell construction shows that the coexistence pressure Π = 0, and the phase transition of polyelectrolyte solution leads to a condensed polymer phase and a fluid phase without polymers. We point out that the coexistence of a condensed polymer phase and a diluted polymer phase is only possible if *p* > *p*_0_ (for parameters used in Figure 7, *p*_0_ is about 8 × 10^–4^).

When the fraction of charged monomers is less than the critical value, i.e., *p* < *p*_0_, the corresponding Maxwell construction indicates that the coexistence pressure is always negative (Π < 0). An example of this is shown in Figure 7b. This scenario is thermodynamically forbidden since a stable osmotic pressure can never be negative for polymer solutions.^17,92^ For this scenario, the phase transition of polyelectrolyte solution can result in only a condensed polymer phase and a fluid phase without polymers. The polymer concentration in the condensed phase is simply given by Π = 0 instead of using the Maxwell construction, and it can be written in the form of eq 23:
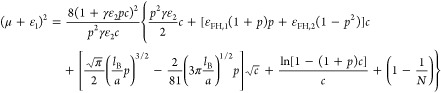
23Equation 23 may have two solutions of *c* for each value of μ, i.e., *c*_1_ → 0 and *c*_2_ → 1, but only the larger one (*c*_2_ → 1) is physically correct. We find that these theoretical considerations were confirmed by the recent experimental observations of the phase behavior of bovine serum albumin proteins in aqueous solutions of the multivalent salt lanthanum chloride (LaCl_3_): At some salt concentrations, only a condensed polymer phase and no dilute polymer phase were observed in reentrant condensation.^28^

By application of the Maxwell construction at the coexistence pressure of Π = 0, we can sketch a parameter space for the coexistence of a dilute and condensed polymer phases in the phase transition of polyelectrolytes. Because the coexistence region defined by the Maxwell construction, as shown in Figure 7a, cannot be obtained analytically in an exact way, we can merely get an approximation for the parameter space. Nevertheless, numerical analysis indicates that the convexity reflection points of osmotic pressure (Π) with respect to monomer concentration (*c*) is an acceptable approximation solution of *c* for Π = 0. In other words, by a combination of Π = 0 (see eq 23 and ∂^2^Π/*∂c*^2^ = 0 for small values of (μ + ε_1_)^2^ when phase separation occurs, i.e., close to the optimally loaded state of the polyelectrolyte with multivalent ions, we get the following approximation for the delineation line when the polyelectrolyte chain is very long (*N* → ∞) and *p* is small:
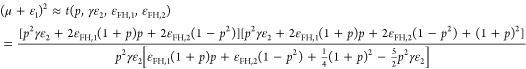
24The details of deriving the above approximation can be found in Appendix C (Supporting Information). To get the above approximation, for simplification we have also ignored the influence of parameter *l*_B_/*a* because in the reasonable range of its experimental values it is unimportant in our model to determine essential behaviors of the reentrant condensation of polyelectrolytes in dilute solutions of multivalent salts. The implications of this simplification are discussed in the following subsection.

**Figure 8:**
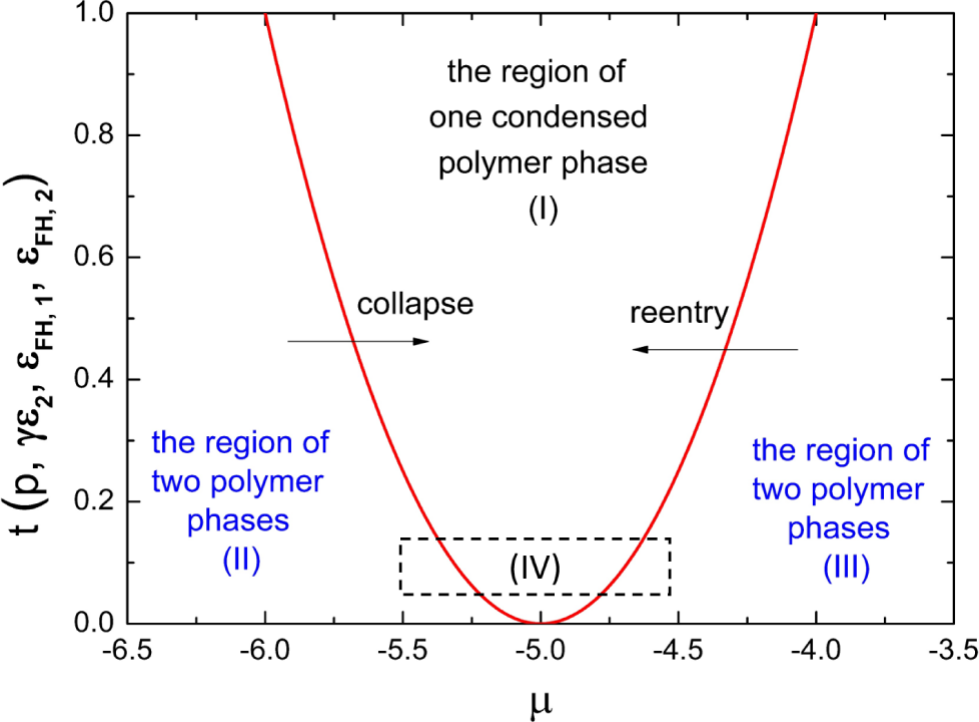
Diagram of the parameter space according to eq 24 toward the coexistence of a dilute and condensed polymer phases in the phase transition of polyelectrolyte, if ε_FH,2_ > 1/2 and the polyelectrolyte chain is very long (*N* → ∞). In the figure, the value of ε_1_ is chosen to be 5.0. By variations of salt concentration in phase transition (such as the region IV in the figure), it is possible to see a change from the coexistence of two polymer phases to the existence of only a condensed polymer phase.

We can read some interesting physics from eq 24. First, the enhancement of the electrostatic gluonic effect (γε_2_) can promote the existence of only one condensed polymer phase in phase transition. This observation can be further checked by a numerical analysis of osmotic pressure even for large values of (μ + ε_1_)^2^ when phase separation occurs, i.e., see eq 21. Second, a decrease of the incompatibility between monomers and solvent (ε_FH,1_ and ε_FH,2_), or an increase of the fraction of charged monomer (*p*), will promote the coexistence of a condensed polymer phase and a diluted polymer phase in the phase transition of polyelectrolyte solution. We find that this prediction is somewhat consistent with existing experimental studies on the phase transition of amphiphilic proteins,^57^ where the concentration of protein in the dilute phase becomes lower when the hydrophobic interaction between protein and water becomes stronger. Third, eq 24 indicates that only a condensed polymer phase exists for uncharged polymers (*p* → 0) if the solvent quality is poor enough, which indeed recovers the result of the classical Flory–Huggins model^66,67^ and can be further seen graphically from Figure 8.

In Figure 8, we display the diagram of the parameter space according to eq 24 toward the coexistence of dilute and condensed polymer phases in the phase transition of polyelectrolytes. According to Figure 8, it also becomes clear that for moderate values of ε_FH,2_ and small *p* and keeping other parameters as constants, it is possible to see a change from the coexistence of two polymer phases to the existence of only a condensed polymer phase by variations of salt concentration in phase transition (such as the region IV in the figure). We note that this prediction concurs with the phase behavior of reentrant condensation of bovine serum albumin proteins in aqueous solutions of multivalent salt lanthanum chloride (LaCl_3_): In the collapse transition, only a condensed polymer phase and no dilute polymer phase were observed at some salt concentrations; while in the reentry transition, the coexistence of two polymer phases was observed at some salt concentrations.^28^

### 3.5 The Effect of Pairwise-Like Electrostatic Interaction from the Long-Range Correlation of All Ions in Reentrant Condensation

The essential feature of our model for the phase separation of polyelectrolytes in the dilute solution of multivalent salts is the induced short-range electrostatic gluonic effect between ionic monomers by forming “physical cross-links” via sharing multivalent ions. An important prediction of our model is that polyelectrolytes cannot play a phase transition, merely because of the nonassociative pairwise-like electrostatic interaction arising from the long-range correlation of all ions in dilute solution. This can be seen clearly from eq 22 by setting γε_2_ = 0. Notice that this prediction concurs with the fact that no phase separation occurs when no multivalent ions exist in dilute solutions of polyelectrolytes (e.g., see a recent excellent monograph by Muthukumar^17^ on this topic).

**Figure 9:**
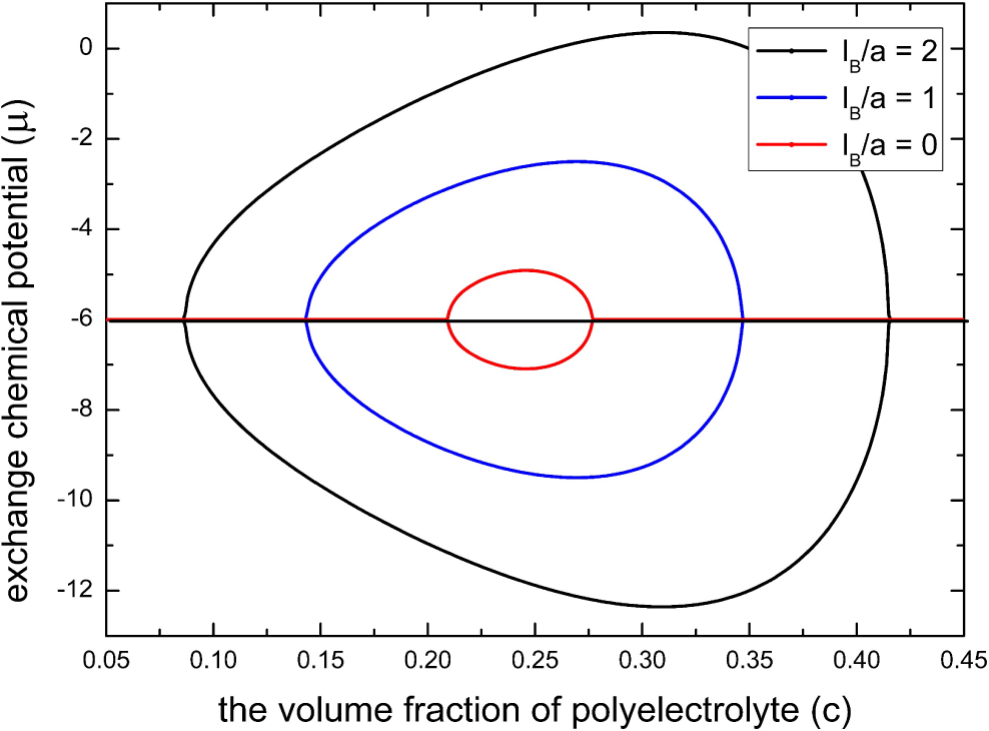
Illustration of the effect of nonassociative pairwise-like electrostatic interactions from the long-range correlation of all ions on the phase behavior of polyelectrolyte solution according to eq 22 by variations of the parameter *l*_B_/*a*. The existence of long-range pairwise-like electrostatic interaction shifts the coexistence region of collapse transition to lower concentrations of multivalent salts but shifts the coexistence region of reentry transition to higher concentrations of multivalent salts. In the figure, the parameters for the spinodal phase diagrams are chosen as infinite chain length (*N* → ∞), *p* = 0.2, ε_FH,1_ = 0.45, ε_FH,2_ = 0.72, ε_1_ = 6, and γε_2_ = 32.

Nevertheless, our analytical results show that the mechanism of short-range electrostatic gluonic effect between ionic monomers, due to forming multivalent ion-assisted “physical cross-links”, can be controlled/interfered by pairwise-like electrostatic interaction arising from the long-range correlation of all ions in dilute solution, which cannot be simply ignored like recent theoretical approaches^93,94^ for phase transition of associative polymers where the small molecule-assisted “physical cross-linking” effect plays trivial roles. In Figure 9, by variations of the parameter *l*_B_/*a* according to eq 22, we illustrate that the existence of pairwise-like electrostatic interaction by the long-range correlation of all ions can shift the coexistence region of collapse transition to lower concentrations of multivalent salts and can shift the coexistence region of reentry transition to higher concentrations of multivalent salts. It is remarkable that this analytical result is in agreement with the seminal results reported by Olvera de la Cruz^95^ and Brilliantov^96−98^ with their coworkers, who showed that a polyelectrolyte chain tends easier to undergo a first-order phase transition in a salt-free polyelectrolyte solution if with a larger value of the parameter *l*_B_/*a*. From Figure 9, we also see that for a state close to the coexistence line, the absence of long-range pairwise-like electrostatic interaction may cause a transition from the condensed to a dissolved polymer phase. It is worth noting that this effect also depends on the chain length of polyelectrolytes. We point out that these observations are manifestations of the fact that the free energy component is negative due to the electrostatic pairwise-like interaction by the long-range correlation of all ions, i.e., eq 5.

### 3.6 The Confluence Effect of Nonelectrostatic Interactions between Solvent and Charged (ε_FH,1_) and Uncharged (ε_FH,1_) Monomers in Reentrant Condensation

The parameter ε_FH,1_ in our model is not essential from purely theoretical consideration. It can in principle be absorbed within the parameter ε_FH,2_, which can be easily seen by redefined uncharged monomers via regrouping the uncharged moieties of polyelectrolyte together. We point out that by variations of ε_FH,1_, the physical conclusions obtained in previous subsections do not change. We thus avoided discussing the effect of parameter ε_FH,1_ in previous subsections for theoretical simplification.

**Figure 10:**
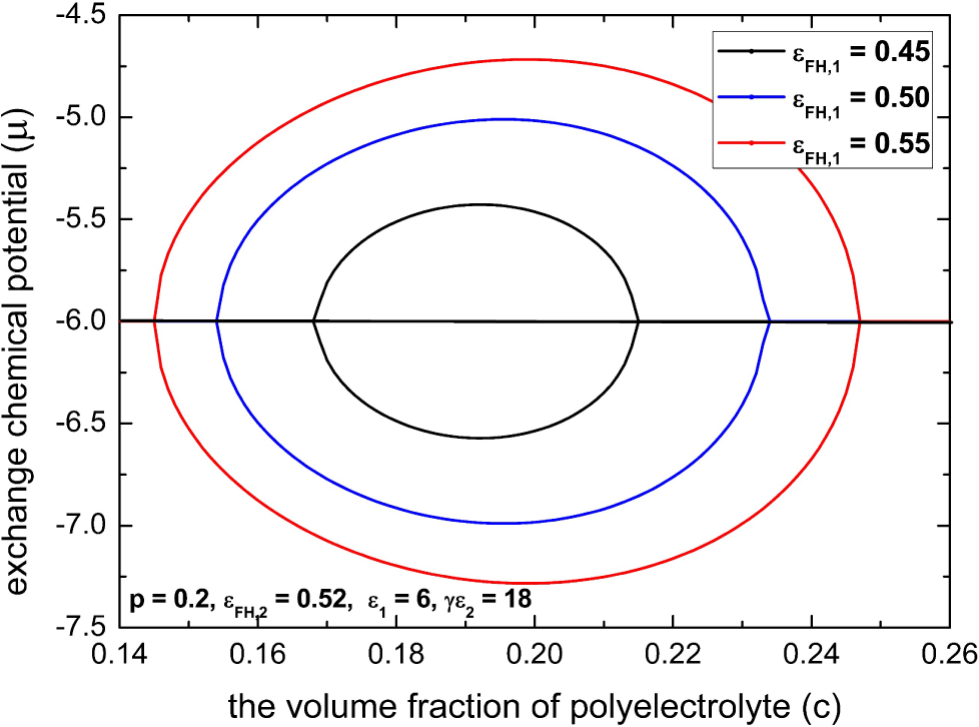
By variations of ε_FH,1_ according to eq 22, an illustration of the confluence effect of nonelectrostatic interactions between solvent (water) and charged (ε_FH,1_) and uncharged (ε_FH,2_) monomers in the multivalent salt-induced reentrant condensation of polyelectrolytes. The parameters for the spinodal phase diagrams are chosen as infinite chain length (*N* → ∞), *l*_B_/*a* = 2.5, *p* = 0.2, ε_FH,2_ = 0.52, ε_1_ = 6, and γε_2_ = 18.

However, it has practical significance for the study of amphiphilic polyelectrolytes such as proteins by separating the parameter ε_FH,1_ from the parameter ε_FH,2_. In Figure 10, we display the confluence effect of nonelectrostatic interactions between solvent (water) and charged (ε_FH,1_) and uncharged (ε_FH,2_) monomers in the multivalent salt-induced reentrant condensation of polyelectrolytes. We see that the confluence effect of parameters ε_FH,1_ and ε_FH,2_ promotes the coexistence region of collapse transition to lower concentrations of multivalent salts and can shift the coexistence region of reentry transition to higher concentrations of multivalent salts. From the example shown in Figure 10, we also note that in a very narrow range of diluted salt solutions, a slight poor-solvent condition for the uncharged moieties (ε_FH,1_ = 0.45, ε_FH,2_ = 0.52) with a rather moderate electrostatic gluonic effect (γε_2_ = 18) is enough for the multivalent salt-induced reentrant condensation of polyelectrolytes to occur. This is in contrast to the cases of large values of γε_2_ shown in previous subsections, and it is common for amphiphilic polyelectrolyte such as proteins.^8,11^

Interestingly, similar to the above discussion, our theoretical approach can be leveraged to explain/analyze certain unusual phase behaviors of highly charged polyelectrolytes. For example, eq 1 indicates and renormalizes that the effective saturated charge density is at the level of *p* ≈ *a*/*l*_B_ for highly charged polyelectrolytes. The parameter ε_FH,2_ ≫ 1/2 (see eq 6) can further roughly take care of the (strong) nonelectrostatic attraction effect (such as the effective hydrophobic attraction)^37,95,99,100^ among these ionic monomers that occurred counterion condensation or not dissociated (with a fraction of about 1 – *p*). The corresponding spinodal phase diagrams according to our theory (similar to Figure 10; also see eq 22) indicate that the coexistence region of the reentry transition shifts significantly to high concentrations of multivalent salts. This explains qualitatively the long-standing problem why it is easy to occur collapse transition but not reentry transition for some highly charged polyelectrolytes if without massive addition of small multivalent salts.^20,37,38,41,49,90,91^

## 4 Conclusions

The motivation of our work was focused on the fact that polyelectrolytes cannot display phase separation merely by the nonassociative pairwise-like electrostatic interactions, due to the long-range correlation of all ions in dilute salt solutions at room temperature.^17^ An important consequence of analogy with the concept of a cosolvent-assisted “physical cross-linking” effect^62,63^ in this work is that we separate the electrostatic effect into the short-range electrostatic gluonic effect due to sharing of multivalent salt ions between ionic monomers and the nonassociative pairwise-like electrostatic effect by the long-range correlation of all ions. This approach allows us to uncover that the electrostatic gluonic effect rather than other effects dominates the reentrant phase transition of the polyelectrolyte in the dilute solutions of multivalent salts. Our theoretical calculations indicate that a minimum coupling energy for the electrostatic gluonic effect is essential for a phase transition to occur. For the first time, this explains the puzzling experimental observation^50−55^ that polyelectrolytes can only show a phase transition in dilute solution of salts with selective multivalency.

In contrast to uncharged linear polymers,^17,19,62,87,88^ a distinctive finding of the present model in this work for the reentrant condensation of polyelectrolytes is that it is hard to realize a real dilute phase when the fraction of charged monomers is not sufficiently low. This is clear from our theoretical calculations for the limiting case of an infinite chain length. Our model justifies that the monomer charge is an important factor that can regulate the polymer liquid–liquid phase separation. Another interesting aspect of our model in the present work is that the strong adsorption between the ionic monomer and multivalent ion can be attributed to the peculiar phenomenon that rather low concentrations of multivalent salts trigger both collapse and reentry transitions of polyelectrolytes. We have also shown theoretically that the incompatibility of the uncharged moieties of polyelectrolytes with water is critical for regulating phase behaviors of polyelectrolytes in aqueous solutions, which is in agreement with recent experimental investigations on solution properties of amphiphilic proteins^56−60^

Nevertheless, it is necessary to point out that a limitation of the current theory is that it has neglected the entropic effect because of the charge distribution on the polyelectrolyte chains. This approach simplifies our analytical calculations but underestimates the overall entropic effect, which may have consequences on the study of biopolyelectrolytes where the charge distribution is an important factor in regulating their phase behaviors.^101^ For example, the charge distribution on globular proteins has a noticeable entropy effect on their liquid–liquid phase separations, which can alter their temperature-dependent phase behaviors in aqueous solutions.^60,102,103^ A starting point of theoretically accounting for the charge distribution of polyelectrolyte chains can be the calculation framework summarized by Avni, Andelman, and Podgornik.^104^ Another limitation of the current theory is that it is primarily confined to dilute solutions of small multivalent salts when reentrant condensation of flexible polyelectrolytes occurs. This means that eq 5 breaks down for medium and concentrated salt solutions, even though the key formalism of eq 4 as an interpolation construction for the short-range electrostatic gluonic effect is still phenomenologically applicable.^33,34^ Now, the effects of electrostatic screening or anomalous underscreening^105^ play important roles, and dipole/multipole-induced Wigner liquid phase^73^ may form. Ways to face this challenge can be by considering the formulation of the “double screening theory”^25,35^ for the crossover regime^106^ of polyelectrolyte solution or by considering the random phase approximation^107^ and the scaling approach^108,109^ for concentrated polyelectrolyte solution. However, a detailed investigation of these above aspects is worthy of future consideration and lies beyond the scope of the present study.

To conclude, motivated by recent all-atom simulation results reported by Wang and coworkers^33,34^ on phase behaviors of polyelectrolyte in aqueous solutions of multivalent salts, and by viewing the multivalent ion as a kind of charged “gluonic cosolute”,^62,63^ in this work, we constructed a simple but effective mean-field model to consistently explain the reentrant condensation of polyelectrolyte in dilute solutions of multivalent salts. We showed that the short-range electrostatic gluonic effect between ionic monomers due to sharing of multivalent salt ions plays a dominant role in governing the phase features of the reentrant condensation. This is reflected in the theory by a nonmonotonic concentration-dependent χ-function (see eq 19). Moreover, we found that the interplay between electrostatic and nonelectrostatic interactions together regulates the phase transition of polyelectrolyte in the dilute solution of multivalent salts. This is reflected by the theory that various solution parameters together control the minimum coupling energy for the electrostatic gluonic effect in phase transition (see eq 12), which is particularly manifested by the rich solution phase behaviors of amphiphilic polyelectrolyte.

## Supporting Information Available

Methodology of finding a spinodal point (Appendix A); methodology of finding an equilibrium state (Appendix B); details of deriving eq 24 (Appendix C) (PDF)

## Footnotes

a The expression “dilute solution of a small salt” in this research means that the Debye–Hückel theory remains qualitatively valid for the small salt, which requires that the ionic strength of the added small salt is low, usually on the order of 200 mmol/L.^78^
