# Supplementary material for: Reentrant Condensation of Polyelectrolytes Induced by Diluted Multivalent Salts: The Role of Electrostatic Gluonic Effects
Source: Biomacromolecules. 2024 Oct 21;25(11):7361–76. doi: 10.1021/acs.biomac.4c01037 (PMC11558675; doi:10.1021/acs.biomac.4c01037)
Supplement: Supplementary file 1 — bm4c01037_si_001.pdf [file bm4c01037_si_001.pdf]

Supporting Information *for*

"Reentrant condensation of polyelectrolytes induced by diluted multivalent salts: the role of electrostatic gluonic effect"

Huaisong Yong[1,2,3*]

[1]Department of Molecules & Materials, MESA+ Institute, University of Twente, 7500 AE Enschede, The Netherlands

[2]Institute Theory of Polymers, Leibniz-Institut für Polymerforschung Dresden e.V., D-01069 Dresden, Germany

[3]School of New Energy and Materials, Southwest Petroleum University, 610500 Chengdu, China

[*]Correspondence: h.yong@utwente.nl (H. Y.); yonghuaisong@gmail.com (H. Y.)

Appendix A: The methodology of finding a spinodal point

Finding spinodal points of the canonical ensemble-like free energy ($G - \prod_0$) is equivalent to find its Saddle points in Mathematics. We often have to calculate its Hessian matrix in a formal way to work out its spinodal points if the free energy ($G - \prod_0$) is a multivariable function [1]. However, we know that the Saddle point for the variable ϕ is 1/2 from the constructed free energy (see **Equation(4)** in the main text). This feature simplifies our calculation. In the present research, we are interested in the case of very diluted solution of multivalent salt ($c_x \rightarrow 0$), it is therefore not necessary to calculate the Hessian matrix of $G - \prod_0$ in a formal way. The calculation of spinodal points then reduces to the computing approach shown in **Section 3.1** in the main text. The computing methodology behind this approach follows closely with **ref.** [2].

Appendix B: The methodology of finding an equilibrium state

The current model is a mean-field formalism under the framework of the isothermal–isobaric ensemble (*NPT* ensemble). Notice that the volume of polyelectrolyte solution is given by $V = N_m a^3/c$ and changes with the monomer concentration (c), where N_m is the total number of monomers in the polyelectrolyte solution and is fixed in our formalism. Because *the free energy per unit of volume* (G) is considered in the current model, the equilibrium solution of the model should be given by minimizing *the total free energy* (VG) with respect to the volume of polyelectrolyte solution (V):

$$0=\left[\frac{\partial(VG)}{\partial V}\right]_{T,N_m}=\left[\frac{\partial\left(\frac{N_m}{c}a^3G\right)}{\partial\left(\frac{N_m}{c}a^3\right)}\right]_{T,N_m}=\left[\frac{\partial(G/c)}{\partial(1/c)}\right]_{T,N_m}=-c\left(\frac{\partial G}{\partial c}\right)_{T,N_m}+G \tag{A1}$$

We also have the following identity equation

$$\left[\frac{\partial(G/c)}{\partial c}\right]_{T,N_m}=\frac{1}{c}\left(\frac{\partial G}{\partial c}\right)_{T,N_m}-\frac{G}{c^2}=-\frac{1}{c^2}\left[\frac{\partial(VG)}{\partial V}\right]_{T,N_m} \tag{A2}$$

Equation (A2) is equivalent to **Equation (A1)** in the minimization of free energy. It is therefore necessary to minimize *the free energy per monomer* (G/c) with respect to the monomer concentration (c) to find the equilibrium state of the polyelectrolyte phase with respect to the bulk solvent phase. The computing methodology behind this minimization approach follows closely with **refs.** [2-5].

Appendix C: The details of deriving the Equation (24)

By ignoring the influence of parameter l_B/a with the manipulation of $\partial^2\Pi/\partial c^2 = 0$ for **Equation (21)** in the main text, it reads:

$$\begin{aligned}0=\frac{\partial^2\Pi}{\partial c^2}=&-\left[\frac{(1+p)}{1-(1+p)c}\right]^2\\&-2\varepsilon_{FH,1}(1+p)p-2\varepsilon_{FH,2}\left(1-p^2\right)-p^2\gamma\varepsilon_2\\&+\frac{p^2\gamma\varepsilon_2}{4}\left(1-2\gamma\varepsilon_2pc\right)\frac{\left(\mu+\varepsilon_1\right)^2}{\left(1+\gamma\varepsilon_2pc\right)^4}\end{aligned} \tag{A3}$$

With the expansion of c-containing terms up to the accuracy of linear terms under the constraint of a small fraction of charged monomers (p) in **Equation (A3)**, it reads:

$$c\approx\frac{\frac{p^2\gamma\varepsilon_2}{4}\left(\mu+\varepsilon_1\right)^2-\left[p^2\gamma\varepsilon_2+2\varepsilon_{FH,1}(1+p)p+2\varepsilon_{FH,2}\left(1-p^2\right)+(1+p)^2\right]}{\frac{3}{2}p^3\left(\gamma\varepsilon_2\right)^2\left(\mu+\varepsilon_1\right)^2+2(1+p)^3} \tag{A4}$$

With the expansion of c-containing terms up to the accuracy of linear terms under the constraint of small p in **Equation (23)** in the main text, by an insertion of **Equation (A4)** into **Equation (23)** in the main text and ignoring the influence of parameter l_B/a when chain length (N) is large and p is small, it reads:

$$\begin{aligned}0\approx&\frac{p^4\left(\gamma\varepsilon_2\right)^2}{32}\left(\mu+\varepsilon_1\right)^4+p^2\gamma\varepsilon_2\left[\frac{5}{4}p^2\gamma\varepsilon_2-\frac{1}{2}\varepsilon_{FH,1}(1+p)p-\frac{1}{2}\varepsilon_{FH,2}\left(1-p^2\right)-\frac{1}{8}(1+p)^2\right]\left(\mu+\varepsilon_1\right)^2\\&+\frac{1}{2}\left[p^2\gamma\varepsilon_2+2\varepsilon_{FH,1}(1+p)p+2\varepsilon_{FH,2}\left(1-p^2\right)\right]\left[p^2\gamma\varepsilon_2+2\varepsilon_{FH,1}(1+p)p+2\varepsilon_{FH,2}\left(1-p^2\right)+(1+p)^2\right]\end{aligned} \tag{A5}$$

This is a quadratic equation of $(\mu + \varepsilon_1)^2$, which can be solved exactly from model parameters p, $\gamma\varepsilon_2$, $\varepsilon_{\mathrm{FH},1}$ and $\varepsilon_{\mathrm{FH},2}$. The physically meaningful solution of **Equation (A5)** is given by

$$\frac{(\mu+\varepsilon_1)^2}{8} \approx \frac{\varepsilon_{FH,1}(1+p)p+\varepsilon_{FH,2}(1-p^2)+\frac{1}{4}(1+p)^2-\frac{5}{2}p^2\gamma\varepsilon_2}{p^2\gamma\varepsilon_2} + \frac{\sqrt{6p^2\gamma\varepsilon_2\left[p^2\gamma\varepsilon_2-\varepsilon_{FH,1}(1+p)p-\varepsilon_{FH,2}(1-p^2)-\frac{3}{2}(1+p)^2\right]+\frac{1}{16}(1+p)^4}}{p^2\gamma\varepsilon_2} \tag{A6}$$

For small values of $(\mu + \varepsilon_1)^2$ when phase separation occurs, i.e., close to the optimally loaded state of the polyelectrolyte with multivalent ions, the **Equation (24)** in the main text is obtained by ignoring the quartic term of $\mu + \varepsilon_1$ in **Equation (A5)**. Here we quote it for convenience:

$$(\mu+\varepsilon_1)^2 \approx t(p,\gamma\varepsilon_2,\varepsilon_{FH,1},\varepsilon_{FH,2}) = \frac{\left[p^2\gamma\varepsilon_2+2\varepsilon_{FH,1}(1+p)p+2\varepsilon_{FH,2}(1-p^2)\right]\left[p^2\gamma\varepsilon_2+2\varepsilon_{FH,1}(1+p)p+2\varepsilon_{FH,2}(1-p^2)+(1+p)^2\right]}{p^2\gamma\varepsilon_2\left[\varepsilon_{FH,1}(1+p)p+\varepsilon_{FH,2}(1-p^2)+\frac{1}{4}(1+p)^2-\frac{5}{2}p^2\gamma\varepsilon_2\right]} \tag{A7}$$

The **Equation (A7)** is an increasing function of parameters $\gamma\varepsilon_2$, $\varepsilon_{\mathrm{FH},1}$ and $\varepsilon_{\mathrm{FH},2}$ within their physically reasonable ranges, but it is a decreasing function of parameter p.

Supporting references

1. Solokhin, M. A.; Solokhin, A. V.; Timofeev, V. S., Phase-Equilibrium Stability Criterion in Terms of the Eigenvalues of the Hessian Matrix of the Gibbs Potential. *Theoretical Foundations of Chemical Engineering* **2002,** *36* (5), 444–446.
2. Sommer, J.-U., Gluonic and Regulatory Solvents: A Paradigm for Tunable Phase Segregation in Polymers. *Macromolecules* **2018,** *51* (8), 3066-3074.
3. Sommer, J.-U., Adsorption–Attraction Model for Co-Nonsolvency in Polymer Brushes. *Macromolecules* **2017,** *50* (5), 2219-2228.
4. Sommer, J.-U.; Merlitz, H.; Schiessel, H., Polymer-Assisted Condensation: A Mechanism for Hetero-Chromatin Formation and Epigenetic Memory. *Macromolecules* **2022,** *55* (11), 4841-4851.
5. Yong, H.; Sommer, J.-U., Cononsolvency Effect: When the Hydrogen Bonding between a Polymer and a Cosolvent Matters. *Macromolecules* **2022,** *55* (24), 11034-11050.
